# Impact of the Excitation Source and Plasmonic Material on Cylindrical Active Coated Nano-Particles

**DOI:** 10.3390/s110909109

**Published:** 2011-09-21

**Authors:** Samel Arslanagic, Yan Liu, Radu Malureanu, Richard W. Ziolkowski

**Affiliations:** 1 Department of Electrical Engineering, Electromagnetic Systems, Technical University of Denmark, Ørsteds Plads, Bldg. 348, Kgs. Lyngby DK-2800, Denmark; E-Mail: s081095@student.dtu.dk; 2 Department of Photonics Engineering, Technical University of Denmark, Ørsteds Plads, Bldg. 343, Kgs. Lyngby DK-2800, Denmark; E-Mail: rm@fotonik.dtu.dk; 3 Department of Electrical and Computer Engineering, University of Arizona, 1230 E. Speedway Blvd. Tucson, AZ 85721, USA; E-Mail: ziolkows@ece.arizona.edu

**Keywords:** core-shell nano-particles, plasmonics, sensors

## Abstract

Electromagnetic properties of cylindrical active coated nano-particles comprised of a silica nano-cylinder core layered with a plasmonic concentric nano-shell are investigated for potential nano-sensor applications. Particular attention is devoted to the near-field properties of these particles, as well as to their far-field radiation characteristics, in the presence of an electric or a magnetic line source. A constant frequency canonical gain model is used to account for the gain introduced in the dielectric part of the nano-particle, whereas three different plasmonic materials (silver, gold, and copper) are employed and compared for the nano-shell layers.

## 1. Introduction

During the past decade considerable efforts have been devoted to the broad field of metamaterials. The areas of interest encompass potential applications spanning from the microwave [1] to the optical frequencies [2]. As regards the latter, among the vast amount of interesting discoveries, notable attention has been devoted to the design of metamaterials and their applications which incorporate active media with plasmonic materials [3,4]. In particular it has been shown that a properly designed active coated nano-particle (CNP) can lead to novel resonance and transparency effects as the intrinsic losses inherent to the plasmonic materials are overcome by suitable gain impregnation of the CNPs. In the majority of the studies of these effects, the CNPs were taken to be of a spherical shape; this was likewise most often the case in several of the previous studies of small particles with gain [5–7].

The properties of the passive coated cylindrical particles in the presence of an arbitrarily located electric line source were thoroughly examined in [8]. The feasibility of a similar configuration to provide resonant active CNPs in the presence of electric as well as magnetic line sources was briefly investigated in a recent work [9]. In there, several super-resonant active CNPs were proposed offering significant enhancements of the radiated power due to a strong excitation of the dipole mode inside the particle. The present work reports an extension of the studies in [9] and includes a more detailed account of the analytical results and, moreover, provides additional numerical results illustrating the super-resonant properties of the investigated CNPs and some of their differences relative to the spherical active CNPs studied in [7]. The CNPs in this work are made of specific dielectric materials, while silver, gold and copper are employed as the plasmonic materials to quantify the amount of gain needed to overcome the losses in the different cases. The gain model employed in the entire analysis is a canonical, single frequency model, and its values are used for different plasmonic materials. The analysis of the cylindrical active CNPs is conducted with a thorough investigation of their near- and far-field properties for altering locations of the ELS. It will clarify the potential application of these CNPs for nano-sensor applications. Specifically, the manuscript is organized as follows: Section 2 introduces the CNP configuration and presents the most important analytical results, while Section 3 discusses the gain and plasmonic material models used. In Section 4 the numerical results are presented and discussed for both electric and magnetic line source illumination of the CNPs. The entire work is summarized and concluded in Section 5. Throughout this work, the time factor exp(*jωt*), with *ω* being the angular frequency and *t* being the time, is assumed and suppressed.

## 2. Configuration and Theory

### 2.1. Configuration

**Figure 1.**
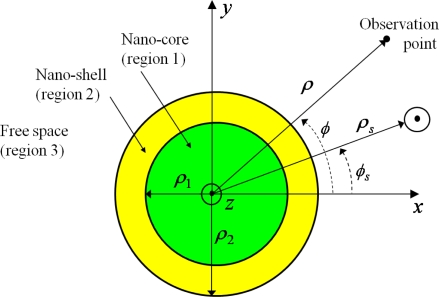
The configuration of a cylindrical CNP excited by either an electric or a magnetic line source.

The CNP configuration is depicted in Figure 1. It consists of a cylindrical nano-core (region 1) with radius *ρ*_1_, covered with a concentric cylindrical nano-shell (region 2) with outer radius *ρ*_2_. The host medium (region 3) of the CNP is that of free-space with the permittivity, *ɛ*_0_, permeability, *μ*_0_, and wave number, $k_{0}=\omega \sqrt{{ɛ}_{0}\mu_{0}}=2\pi /\lambda$, where *λ* is the free space wavelength. The CNP is excited by an arbitrarily located line source for which two polarizations are considered: the TM (with respect to *z*) polarization, in which case the source is an electric line source (ELS) with constant electric current *I_e_* [A/m], and the TE (with respect to *z*) polarization, in which case the source is a magnetic line source (MLS) with constant magnetic current *I_m_* [V/m]. The two regions 1 and 2 consist of simple, lossy materials with the permittivity, *ɛ_i_* = *ɛ′_i_* − *jɛ″_i_*, permeability, *μ_i_* = *μ*_0_, and wave number $k_{i}=\omega \sqrt{{ɛ}_{i}\mu_{0}}$, *i* = 1 and 2, where the branch of the square root will be discussed below. A cylindrical coordinate system (*ρ*, *ϕ*, *z*) and an associated rectangular coordinate system (*x*, *y*, *z*) are introduced such that their origins coincide with the center of the CNP. The coordinates of the observation point are (*ρ*, *ϕ*), and those of the line source are (*ρ_s_*, *ϕ_s_*).

### 2.2. Theory

The analytical solution to the problem depicted in Figure 1 is rather straightforward to obtain and it has been outlined in detail in [8]. For the purposes of the present work we only present its main points. For both polarizations, the field due to the line source constitutes the known incident field and it is expanded in terms of cylindrical wave functions. The unknown fields due to the CNP in the three regions are likewise expanded in terms of cylindrical wave functions and they involve the unknown expansion coefficients $C_{i,n}^{\mathrm{TM}}$ for TM polarization, and $C_{i,n}^{\mathrm{TE}}$ for TE polarization. For both sets of coefficients, *i* = 1 for the field in region 1, *i* = 2 and 3 for the field in region 2, and *i* = 4 for the field in region 3, while the symbol *n* is the mode number with *n* = 0 referring to the monopole mode in the expansion, *n* = 1 to the dipole mode, *etc*. for the other modes. The unknown expansion coefficients depend on the location of the line source, and are easily obtained by enforcing the boundary conditions on the two cylindrical interfaces, *ρ* = *ρ*_1_ and *ρ* = *ρ*_2_, for all values of *ϕ*.

For the purpose of our investigations of the electromagnetic properties of the CNP excited by an ELS or a MLS, the normalized radiation resistance (NRR) is examined along with the spatial distribution of the electric and magnetic fields as well as the directivity patterns. The NRR is the ratio of the radiation resistance of the line source in the presence of the CNP to its value in the absence of the CNP. In particular, with *P_CNP_* representing the total average power radiated by either the ELS or MLS in the presence of the CNP, the corresponding radiation resistance, *P_CNP_*, is defined by:

(1a) $$R_{\mathrm{CNP}}=\frac{2P_{\mathrm{CNP}}}{I^{2}}$$

for a given constant value of the current *I* along the line source, where *I* = *I_e_* (*I_m_*) for TM (TE) polarization. In a similar manner, the radiation resistance, *R_LS_*, of either the ELS or MLS radiating alone in free space for the same current *I* along the source is defined by:

(1b) $$R_{\mathrm{LS}}=\frac{2P_{\mathrm{LS}}}{I^{2}}$$

with *P_LS_* representing the total average power radiated by either the ELS or MLS alone in free space. It is noted that the explicit expressions for *P_CNP_* and *P_LS_* can be found in [8] for the TM polarization case. In mathematical terms, the NRR is thus given by:

(2a) $$\text{NRR}\equiv \frac{R_{\mathrm{CNP}}}{R_{\mathrm{LS}}}=\frac{1}{2}\sum_{n=0}^{N_{\text{max}}}\tau_{n}^{2}(3-\tau_{n}){\left|\alpha_{n}\right|}^{2}$$

where

(2b) $$\alpha_{n}=\{\begin{array}{cc} C_{4,n}^{\mathrm{TM}} & \text{ELS in region}\;1\;\text{and}\;2\\ J_{n}(k_{0}\rho_{s})+C_{4,n}^{\mathrm{TM}} & \text{ELS in region}\;3 \end{array}$$

for TM polarization, and:

(2c) $$\alpha_{n}=\{\begin{array}{cc} C_{4,n}^{\mathrm{TE}} & \text{MLS in region}\;1\;\text{and}\;2\\ J_{n}(k_{0}\rho_{s})+C_{4,n}^{\mathrm{TE}} & \text{MLS in region}\;3 \end{array}$$

for TE polarization. In Equations (1) and (2), *J_n_*(*k*_0_*ρ_s_*) is the Bessel function of order *n*, *τ_n_* is the Neumann number, *i.e.*, *τ_n_* = 1 for the *n* = 0 mode and *τ_n_* = 2 otherwise, while *N*_max_ is the truncation limit in the implementation of the exact infinite summation and is chosen to ensure the convergence of the expansion in Equation (1).

The directivity, *D*, defined as the ratio of the radiation intensity to the total average power per unit angle, can be expressed as:

(2d) $$D(\phi )=\frac{2\cdot {\left|\sum_{n=0}^{N_{\text{max}}}\tau_{n}j^{n}\alpha_{n}\;\text{cos}[n(\phi -\phi_{s})]\right|}^{2}}{\sum_{n=0}^{N_{\text{max}}}\tau_{n}^{2}(3-\tau_{n}){\left|\alpha_{n}\right|}^{2}}$$

## 3. Gain and Material Models

The present work examines three different CNPs. For each of them, region 1, the cylindrical nano-core, is composed of silica-oxide (SiO_2_) while three different plasmonic materials are considered for region 2, the nano-shell: silver (Ag), gold (Au) and Copper (Cu). The corresponding structures are referred to as the Ag-, Au-, and Cu-based cylindrical CNPs. The radius of the nano-core for all CNPs is set to *r*_1_ = 24 nm, while the outer radius of the nano-shell is set to *r*_2_ = 30 nm, resulting in a 6 nm thick plasmonic nano-shell. This choice matches the spherical active CNP cases considered in [3,4,7].

The permittivity, *ɛ*_1_, of the silica nano-cylinder is comprised of a contribution from its refractive index in the frequency region of interest ( $n=\sqrt{2.05}$) and a contribution from the canonical gain model. It is thus expressed as:

(3) $${ɛ}_{1}={ɛ}_{0}(n^{2}-\kappa^{2}-j2\mathrm{nk})$$

where *κ* determines the nature of the nano-cylinder which is lossless and passive for *κ* = 0, lossy and passive for *κ* > 0, and active for *κ* < 0. Thus the amount of gain introduced in the CNP configuration to overcome the plasmonic material losses is tailored by the choice of the parameter *κ*.

**Figure 2.**
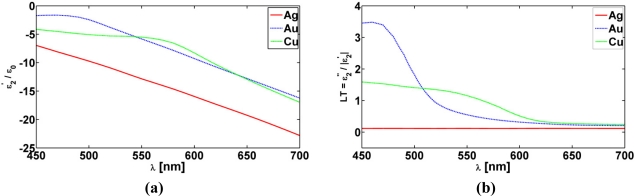
(**a**) The real, *ɛ*_2_′, part of the 6 nm thick Au, Ag, and Cu nano-shells normalized to the free-space permittivity *ɛ*_0_, and (**b**) the corresponding loss tangents LT = *ɛ*_2_″/|*ɛ*_2_′|, as functions of the excitation wavelength *λ*.

As to the permittivity, *ɛ*_2_, of the plasmonic nano-shell we note that its size dependency must be taken into account due to the nano-scale dimension of the CNPs. To this end, empirically determined bulk values of Ag, Au and Cu permittivities have been employed [3] and their real parts, *ɛ*_2_′, normalized with the free-space permittivity *ɛ*_0_, are shown in Figure 2 for a 6 nm thick Ag, Au, and Cu nano-shell along with the associated values of their loss tangents defined by LT = *ɛ*_2_″/|*ɛ*_2_′| as functions of the excitation wavelength.

As observed in Figure 2, the real part of the permittivity of the various plasmonic materials under consideration is negative in the depicted wavelength range, and moreover that they all are dispersive and lossy with Ag being the least lossy case.

## 4. Results and Discussion

We first present and discuss the results pertaining to the TM polarization cases. Subsequently, those for the TE polarization cases are discussed. Throughout the following numerical investigations, the line currents of the corresponding sources are set to *I_e_* = 1 [A/m] for the ELS and *I_m_* = 1 [V/m] for the MLS.

### 4.1. TM Polarization

**Figure 3.**
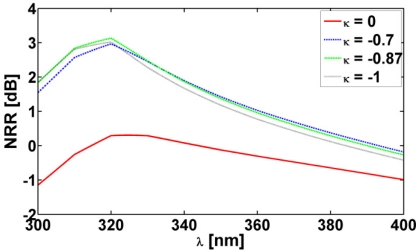
The NRR of the Ag-based CNPs as a function of the excitation wavelength, *λ*, for different values of the parameter *κ* for TM polarization. The ELS is in region 1 at (*ρ_s_*, *ϕ_s_*) = (12 nm, 0°).

Figure 3 shows the NRR as a function of the excitation wavelength, *λ*, for the Ag-based CNP for different values of the parameter *κ*. The ELS is located in region 1 at (*ρ_s_*, *ϕ_s_*) = (12 nm, 0°). It is rather obvious that for this particular polarization, no resonance, *i.e*., no large values of the NRR, is in evidence, regardless of the value of *κ*. The quantity NRR ≈ 0.3 dB for *κ* = 0 at *λ* = 324.6 nm, while NRR ≈ 3.13 dB for *κ* = −0.87 at *λ* = 320 nm. This is an expected result since the resonance condition [8,10]:

(4) $$\frac{\rho_{1}}{\rho_{2}}\approx \sqrt[{2n}]{\frac{\left({\mu^{\prime }}_{2}+{\mu^{\prime }}_{1})({\mu^{\prime }}_{2}+\mu_{0}\right)}{\left({\mu^{\prime }}_{2}-{\mu^{\prime }}_{1})({\mu^{\prime }}_{2}-\mu_{0}\right)},}n\geq 1$$

holds for the corresponding small lossless structure of Figure 1 in the case of TM polarization. As shown in [8,10], at least one of the parameters, *μ*_1_′ or *μ*_2_′, must be negative to satisfy Equation (4) and thus provide large NRR values. However, since for the CNP in question, as well as for the remaining two CNPs, the permeabilities of all regions are those of free-space, no resonance and, thus, no significant enhancement of the NRR occurs for this particular polarization.

**Figure 4.**
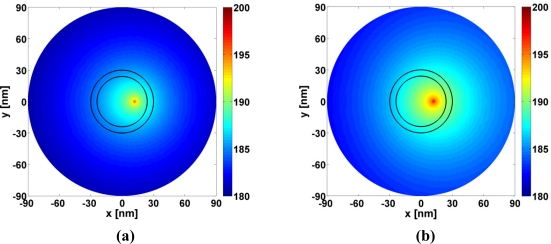
The magnitude of the electric field of the Ag-based CNP for TM polarization when (**a**) *κ* = 0, *λ* = 324.6 nm, and (**b**) *κ* = −0.87, *λ* = 320 nm. The ELS is located in region 1 at (*ρ_s_*, *ϕ_s_*) = (12 nm, 0°). The field is shown in a circular region with radius of 90 nm. The curves representing the cylindrical surfaces of the CNP are likewise shown in the figure. In terms of the distance normalized by the free space wavelength, *λ*, the *x*- and *y*-axes in (a) and (b) span the intervals [–0.277; +0.277] *λ* and [–0.281; +0.281] *λ*, respectively.

These conclusions are further illustrated with Figure 4 which shows the quantity 20 log_10_ |*E⃗*(*ρ*, ∅)|, where *E⃗*(*ρ*, ∅) is the total electric field normalized by 1 V/m, in a circular region of radius 90 nm for the Ag-based CNP for *κ* = 0 and *λ* = 324.6 nm [Figure 4(a)] and *κ* = −0.87, *λ* = 320 nm [Figure 4(b)]. In both cases, the field is dominated by that of the ELS alone, *i.e.*, the CNP does not exhibit any influence on the ELS or the local field distribution. Although not shown, similar results were found for the Au- and Cu-based CNPs.

### 4.2. TE Polarization

**Figure 5.**
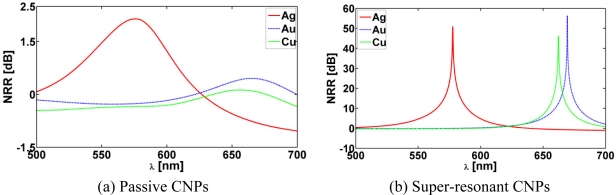
The NRR as a function of the excitation wavelength, *λ*, of passive (**a**) and super-resonant (**b**) Ag-, Au-, and Cu-based CNPs, when the MLS is in region 1 at (*ρ_s_*, *ϕ_s_*) = (12 nm, 0°).

Figure 5 shows the NRR as a function of the excitation wavelength, *λ*, for the three CNPs with (a) *κ* = 0 and (b) the corresponding super-resonant states which occur with *κ* = −0.175, *κ* = −0.262 and *κ* = −0.310, respectively, for the Ag-, Au-, and Cu-based CNPs. In all cases, the MLS is in region 1 at (*ρ_s_*, *ϕ_s_*) = (12 nm, 0°). For the super-resonant states the NRR values are significantly increased and the intrinsic losses of the plasmonic materials are vastly overcome relative to the passive CNP results reported in Figure 5(a). The NRR values, as well as the values of the parameter *κ* needed for the super-resonance to occur, are summarized in Table 1 for the three CNPs along with the values of the wavelength *λ* at which the respective resonances are attained. From Table 1 it follows that the magnitude of the parameter *κ* needed to attain the super-resonant states is largest for the Cu-based CNP. This is an expected result since copper has the highest losses of the three plasmonic materials utilized in the design of present CNPs, cf., Figure 2(b).

**Figure 6.**
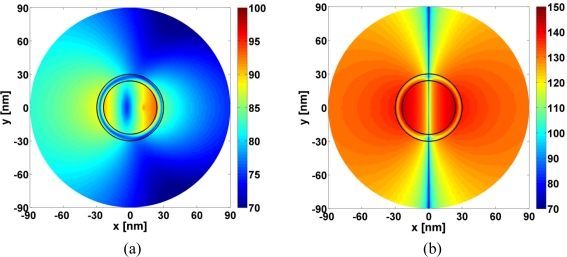
The magnitude of the magnetic field of the Ag-based CNP for the TE polarization when (**a**) *κ* = 0, *λ* = 575.69 nm, and (**b**) *κ* = −0.175, *λ* = 577.70 nm. The MLS is located in region 1 at (*ρ_s_*, *ϕ_s_*) = (12 nm, 0°). The field is shown in a circular region with radius of 90 nm. The curves representing the cylindrical surfaces of the CNP are likewise shown in the figure. Note that the dynamic range in (b) is larger than in (a). In terms of the distance normalized to the free space wavelength, *λ*, the *x*- and *y*-axes in (a) and (b) span the interval [–0.156; +0.156] *λ*.

The super-resonances reported in Figure 5(b) are due to a strong excitation of the dipole mode in the respective CNPs. This is confirmed in Figure 6(b) which shows the spatial distribution of the total magnetic field (more specifically, the quantity 20 log_10_ |*H⃗*(*ρ*, ∅)|), where *H⃗*(*ρ*, ∅) is the total magnetic field normalized by 1 A/m, for the Ag-based CNP with *κ* = −0.175 and *λ* = 577.70 nm, where a clear and strong dipolar field distribution is in evidence. In contrast, the magnetic field of the corresponding passive Ag-based CNP (*κ* = 0) reported in Figure 6(a) is a mixture of monopole and dipole modes. This configuration clearly is insufficient to provide enhancement of the NRR. These results are in agreement with the results of Figure 5.

Although not shown, the magnetic field distributions for the Au- and Cu-based CNPs in their super-resonant states resemble that of the Ag-based CNP, *i.e.*, the large NRR values for these CNPs are likewise due to the excitation of the resonant dipole mode inside the CNPs. It is interesting to remark that these results are in line with the resonance condition [8,10]:

(5) $$\frac{\rho_{1}}{\rho_{2}}\approx \sqrt[{2n}]{\frac{\left({{ɛ}^{\prime }}_{2}+{{ɛ}^{\prime }}_{1})({{ɛ}^{\prime }}_{2}+{ɛ}_{0}\right)}{\left({{ɛ}^{\prime }}_{2}-{{ɛ}^{\prime }}_{1})({{ɛ}^{\prime }}_{2}-{ɛ}_{0}\right)}},n\geq 1$$

which holds for the corresponding small lossless structure of Figure 1 in the case of TE polarization. As shown in [8] and [10], at least one of the parameters, *ɛ*_1_′ or *ɛ*_2_′, must be negative to satisfy Equation (5) and thus provide large NRR values. This requirement can be fulfilled here due to the negative values of *ɛ*_2_′ of the employed plasmonic materials, cf., Figure 2(a).

**Figure 7.**
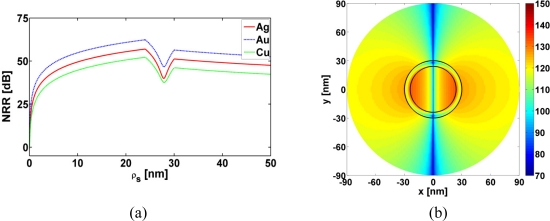
TE polarization results. (**a**) NRR as a function of the MLS location, *ρ_s_*, of the super-resonant Ag-, Au-, and Cu-based CNPs. (**b**) The magnetic field of the Ag-based CNP for *κ* = −0.175, *λ* = 577.70 nm. The MLS is located in region 2 at (*ρ_s_*, *ϕ_s_*) = (27.84 nm, 0°), this being the location at which the minimum NRR is attained in (a). The field is shown in a circular region with radius of 90 nm. The curves representing the cylindrical surfaces of the CNP are shown in the figure. In terms of the distance normalized to the free space wavelength, *λ*, the *x*- and *y*-axes in (b) span the interval [–0.156; +0.156] *λ*.

The resonant behavior of the three examined CNPs is not restricted to the MLS in region 1, but also occurs when the MLS is located in region 2 and 3 as shown in Figure 7(a), where the NRR is shown as a function of the MLS location, *ρ_s_*, for the three super-resonant CNPs. With no enhancements occurring when the MLS is at and very close to the origin, rather large values of the NRR result occur when it is close to the inner surface of the plasmonic shells. Moreover, a dip in the NRR is noted in region 2 for all cases. At these locations the excited resonant dipole mode is somewhat weaker than that shown in Figure 6(b). This is illustrated by Figure 7(b) which shows the magnetic field for the MLS location (*ρ_s_* = 27.84 nm) of the minimum NRR in region 2 for the super-resonant Ag-based CNP.

**Figure 8.**
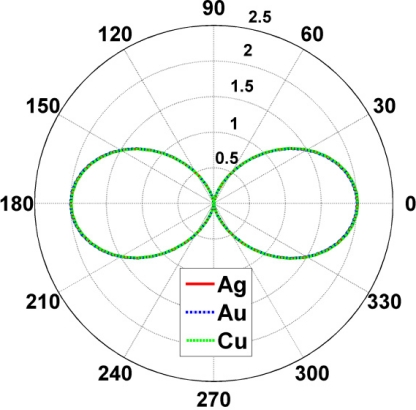
The directivity of the super-resonant Ag-, Au-, and Cu-based CNPs. The MLS is inside the CNPs at (*ρ_s_*, *ϕ_s_*) = (12 nm, 0°). The same results are obtained for the MLS outside the CNPs at (*ρ_s_*, *ϕ_s_*) = (31 nm, 0°).

Having discussed the near-field and the NRR results for a number of active CNPs, we next briefly illustrate their far-field radiation characteristics in terms of their directivity patterns. These are shown in Figure 8 for the super-resonant Ag-, Au-, and Cu-based CNPs when the MLS is inside the CNPs at (*ρ_s_*, *ϕ_s_*) = (12 nm, 0°). When the MLS is outside the CNPs at (*ρ_s_*, *ϕ_s_*) = (31 nm, 0°), the same result is obtained. For both locations of the MLS, all three CNPs lead to exactly the same directivity patterns, *i.e.*, they are symmetric dipolar patterns. Moreover, they all have a maximum directivity of 2. These far-field behaviors agree with the corresponding near-field results. This also means that the super-resonances reported in Figure 5(b) are indeed due to a strong excitation of the dipole mode in the respective CNPs, as noted earlier.

**Figure 9.**
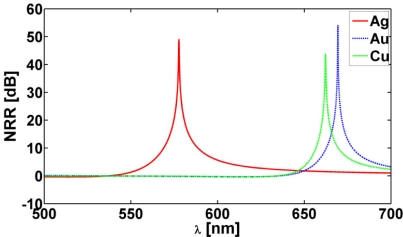
The NRR as a function of the excitation wavelength, *λ*, for super-resonant Ag-, Au-, and Cu-based CNPs for TE polarization. In all cases, the MLS is outside the CNPs at (*ρ_s_*, *ϕ_s_*) = (40 nm, 0°).

The supe-resonant properties of a number of cylindrical active CNPs excited by a MLS have so far been examined. With this in mind, it is very interesting to note a few differences between these CNPs and the correspondingly sized spherical active CNPs studied thoroughly in [7] for the case of an electric Hertzian dipole excitation. In order for the super-resonances to occur lower magnitude values of the parameter *κ* are needed in the cylindrical active CNPs than in the spherical ones, *i.e*., a lower amount of gain is needed in the former than in the latter CNP configurations in order to overcome the intrinsic plasmonic losses. Moreover, the super resonances occur at larger wavelengths for the cylindrical CNPs than for the spherical CNPs, and they result in significantly lower NRR values for the former. These behaviors would have significant impact on any nano-sensor application. Another interesting and important difference between the two geometries is the inability of the cylindrical CNPs to provide reduced NRR values for the examined range of parameters. In [7] it was shown that for the source location outside the super-resonant spherical CNPs, both enhanced as well as reduced NRR values can be obtained. However, as Figure 9 demonstrates, when the MLS is outside the Ag-, Au-, and Cu-based CNPs at (*ρ_s_*, *ϕ_s_*) = (40 nm, 0°), only the super-resonances and no reductions in the NRRs are in evidence; similar results are obtained for other locations of the MLS outside the CNPs.

## 5. Conclusions

Electromagnetic properties of infinitely long cylindrical CNP configurations comprised of a cylindrical silica nano-core covered with a plasmonic nano-shell were investigated in the present work. The source of excitation was taken to be an electric or a magnetic line current, while three different plasmonic materials were employed for the nano-shells, namely silver, gold and copper.

While no resonant phenomenon was observed for the case of the electric line source illumination, for the passive as well as active configurations, it was demonstrated that the inclusion of gain in the silica nano-core significantly helps to overcome the intrinsic losses of the plasmonic nano-shells for the case of magnetic line source illumination. In particular, super-resonant states were identified for the silver-, gold-, and copper-based CNPs and their large enhancements of the normalized radiation resistance were quantified. These enhancements were shown to be due to a strong excitation of the dipole mode inside the respective particles, and were largest for the source locations near-by the inner surface of the nano-shells, while being heavily diminished for source locations near to the center of the particles, in agreement with similar findings for the purely passive cases [8]. The far-field radiation patterns further confirmed this dipole mode behavior. The amount of gain required in the three cases was different and was found, as expected, to be largest for the most lossy material. The existence of the super-resonances for the case of the magnetic line source illumination and their absence for the electric line source case was found to be in agreement with the predictions of certain conditions for resonances derived in literature for electrically small metamaterial-based structures. Moreover, the present results provided the basis for an interesting comparison between the currently investigated cylindrical active coated nano-particles and the corresponding spherical ones studied thoroughly in [7]. For the super-resonances to occur a lower amount of gain is needed in the cylindrical than in the spherical cases; the super resonances for the former structures occur at larger wavelengths and result in lower enhancements for the examined range of parameters. In addition, the cylindrical active CNPs were found only to lead to significant enhancements of the radiated power for magnetic source locations outside the CNPs, whereas the corresponding spherical CNPs are known to lead to both enhanced as well as reduced radiation effects as the source is moved outside the CNPs. The illustrated enhancements of the source fields for the TE polarization could have significant impact on their use for nano-sensor applications.

## Figures and Tables

**Table 1.** The values of the NRR, parameter *κ* and the wavelength *λ* for the super-resonant Ag-, Au-, and Cu-based CNPs for TE polarization. In all cases the MLS is in region 1 at (*ρ_s_*, *ϕ_s_*) = (12 nm, 0°).

**Parameter**	**Ag**	**Au**	**Cu**
NRR [dB]	51.06	56.39	46.26
*κ*	−0.175	−0.262	−0.310
*λ* [nm]	577.70	669.39	662.19
